# Disruption of p-AKT/ERβ-Mediated Midkine Signaling Promotes the Antimetastatic Effects of Timosaponin AIII in Nasopharyngeal Carcinoma Cells

**DOI:** 10.3390/cells15211989

**Published:** 2026-10-31

**Authors:** Chu-Che Lee, Yong-Syuan Chen, Shun-Fa Yang, Chu-Liang Lin, Yi-Hsien Hsieh, Chun-Yi Chuang

**Affiliations:** 1 Institute of Medicine, Chung Shan Medical University, Taichung 40201, Taiwan; dm731849@tzuchi.com.tw (C.-C.L.); ysf@csmu.edu.tw (S.-F.Y.); like751019@gmail.com (C.-L.L.); 2 Department of Medicine Research, Buddhist Dalin Tzu Chi Hospital, Chiayi 62247, Taiwan; 3 Department of Medical Research, Chung Shan Medical University Hospital, Taichung 40201, Taiwan; cshe1470@csh.org.tw; 4 School of Medicine, Chung Shan Medical University, Taichung 40201, Taiwan; 5 Department of Otolaryngology, Chung Shan Medical University Hospital, Taichung 40201, Taiwan

**Keywords:** nasopharyngeal carcinoma, timosaponin AIII, metastasis, MDK, p-AKT, ERβ

## Abstract

**Background**: Nasopharyngeal carcinoma (NPC) is a highly metastatic epithelial malignancy with limited therapeutic options for advanced disease. Timosaponin AIII (TSAIII), a natural steroidal saponin, exhibits antitumor activity; however, its effects on NPC progression and the molecular mechanisms remain unclear. **Method**: Cell viability, proliferation, apoptosis, migration, and invasion were evaluated using cell growth, colony formation, Annexin V/PI, and Transwell assays. Human chemokine array and The Cancer Genome Atlas (TCGA) analyses identified midkine (MDK) as a potential molecular target of TSAIII and its clinical relevance in NPC. Western blotting, qRT-PCR, immunofluorescence, and chromatin immunoprecipitation (ChIP) assays were used to investigate the underlying signaling mechanisms. Antimetastatic activity was further evaluated in C.B17-SCID mice. **Results**: TSAIII suppressed NPC cell migration and invasion with minimal cytotoxicity, accompanied by reduced the expression of p-AKT (Ser473), estrogen receptor β(ERβ) nuclear localization, and MDK expression. Wortmannin enhanced these effects, while TSAIII attenuated latent membrane protein 1 (LMP1)-induced signaling. In vivo, TSAIII significantly reduced lung metastasis without apparent toxicity. **Conclusions**: TSAIII suppresses NPC metastasis by targeting the LMP1/p-AKT/ERβ/MDK signaling axis, supporting its potential as an antimetastatic therapeutic candidate for NPC.

## 1. Introduction

Nasopharyngeal carcinoma (NPC), a malignant tumor arising from the epithelial lining of the nasopharynx, has distinct geographical and ethnic distribution patterns that include Southeast Asia and Southern China [1]. The etiology of NPC is multifactorial, including genetic susceptibility, Epstein–Barr virus (EBV) infection, and environmental factors such as the consumption of salted fish and exposure to wood dust [2,3]. NPC is characterized by its aggressive behavior, early lymphatic spread, and frequent distant metastasis, which significantly complicate treatment [4]. Latent membrane protein 1 (LMP1), an oncogenic protein encoded by EBV, plays a central role in the pathogenesis of NPC [5]. Acting as a constitutively-active receptor that mimics CD40 signaling, LMP1 activates multiple downstream pathways, including NF-κB, MAPK, and PI3K/AKT, that collectively promote cell proliferation, inhibit apoptosis, increase invasion, and facilitate immune evasion. LMP1 expression correlates strongly with NPC tumor progression, metastasis, and poor patient prognosis [6,7].

Midkine (MDK) is a heparin-binding growth factor that is normally expressed during embryogenesis [8]. MDK overexpression promotes tumor progression by increasing cell proliferation, angiogenesis, and metastasis and modulating the immune microenvironment [9]. In NPC, elevated MDK expression has been linked to increased microvessel density and poorer overall survival. Notably, exosomal miR-9 from NPC cells can suppress angiogenesis by targeting MDK and regulating the PDK/AKT signaling pathway in endothelial cells, highlighting the role of MDK in tumor angiogenesis [10]. Previous studies have demonstrated that estradiol promotes ERβ recruitment to the MDK promoter, enhancing MDK transcription and epithelial–mesenchymal transition in lung adenocarcinoma cells [11]. Emerging evidence indicates potential crosstalk between LMP1 and MDK within the NPC tumor microenvironment. LMP1-induced signaling may upregulate factors such as MDK, thereby promoting angiogenesis and tumor aggressiveness through overlapping pathways such as PI3K/AKT [10,12].

Timosaponin AIII (TSAIII), a steroidal saponin predominantly isolated from the rhizomes of Anemarrhena asphodeloides, is a traditional medicinal herb used in East Asian medicine for its antipyretic and anti-inflammatory properties [13,14]. Recent investigations have revealed its significant anticancer properties across various tumor types, including breast, liver, and colorectal cancers [15,16,17]. The anticancer potential of TSAIII has been attributed to multiple mechanisms, including the induction of apoptosis, inhibition of cell proliferation, suppression of angiogenesis, and modulation of key oncogenic signaling pathways such as PI3K/Akt, MAPK, and NF-κB [18,19,20]. These findings show that TSAIII is a promising natural compound with potential therapeutic applications in cancer treatment. However, its effects and molecular mechanism on NPC cells remain poorly understood. Therefore, this study aimed to evaluate the inhibitory effects of TSAIII on NPC cell migration and invasion in vitro and to investigate the potential involvement of the LMP1/p-AKT/ERβ/MDK signaling pathway in these effects. Furthermore, we assessed the antimetastatic efficacy and safety of TSAIII treatment in vivo.

## 2. Materials and Methods

### 2.1. Reagents and Chemicals

Timosaponin AIII (TSAIII; CFN98151) was obtained from ChemFaces (Wuhan, Hubei, China). MTT (2298-93-1) was purchased from GoldBio (St. Louis, MO, USA), fetal bovine serum (FBS; 35-010-CV), Matrigel matrix (354234) and penicillin–streptomycin (30-002-CI) were obtained from CORNING (Corning, NY, USA). Annexin V & Dead Cell Assay Kit was purchased from CYTEK Biosciences (Austin, TX, USA). TRIzol reagent was purchased from EBL Biotechnology (Taipei City, Taiwan). Antibodies against ERα (sc-8002), ERβ (sc-390243), p-AKT-Ser473 (sc-81433), t-AKT (sc-56878), β-actin (sc-47778) were purchased from Santa Cruz Biotechnology (Dallas, TX, USA). Antibodies against p-AKT-Thr308 (NB100-81778) were purchased from Novus Biologicals (Centennial, CO, USA), Antibodies against MDK (A0251) were purchased from ABclonal (Woburn, MA, USA). Antibodies against LMP1 (ab78113) were purchased from Abcam (Cambridge, MA, USA).

### 2.2. NPC Cell-Culture Conditions

Human nasopharyngeal carcinoma (NPC) cell lines (NPC-BM and NPC-39) were a gift from Dr. Ming-Ju Hsieh, Oral Cancer Research Center, Changhua Christian Hospital (Changhua, Taiwan). NPC-39 is an EBV-negative cell line derived from a patient with nasopharyngeal carcinoma. NPC-BM is an EBV-negative and bone marrow-derived metastatic NPC cell line, characterized by enhanced migratory and invasive capabilities. Mycoplasma contamination was assessed using the MycoAlert Mycoplasma Detection Kit (Lonza, Basel, Switzerland). NPC-BM and NPC-39 were cultured in RPMI-1640 medium supplemented with 10% FBS, sodium bicarbonate, and penicillin–streptomycin. Cells were maintained at 37 °C in a humidified incubator containing 5% CO_2_. For the gene transfection assay, an AKT overexpression plasmid (Ov-AKT, 0.5 μg) and the Lipofectamine 3000 transfection reagent (Thermo Fisher Scientific, Carlsbad, CA, USA) were mixed in serum-free medium and incubated for 5 min at room temperature. The transfection mixture was then added to NPC-BM cells and incubated for 6 h. Subsequently, the cells were treated with TSAIII (5 μM) for an additional 24 h. After treatment, the cells were harvested, and the protein expression levels of p-AKT and MDK were analyzed by Western blotting.

### 2.3. Cell Growth Assay

NPC-BM and NPC-39 cells were treated with TSAIII (0, 1, 2, 3, 4, and 5 μM) for 24 or 48 h, followed by the addition of 0.2 mL MTT reagent (0.5 mg/mL in culture medium) and incubation for 4 h. The resulting formazan crystals were dissolved in isopropanol for 10 min and the absorbance (570 nm) measured using a microplate ELISA reader (BioTek Instruments, Winooski, VT, USA). The cell growth rate was determined using the following formula: Cell growth rate (%) = (TSAIII-treated cells/untreated cells) × 100.

### 2.4. MDK ELISA Assay

The secretion levels of MDK were measured using a Human MDK ELISA Kit (FineTest Biotech Inc., Boulder, CO, USA) according to the manufacturer’s instructions. Briefly, TSAIII-treated NPC cells and standards were added to 96-well plates pre-coated with MDK antibodies for 90 min at 37 °C. Subsequently, biotin-labeled antibody working solution was added and incubated for 60 min. After washing to remove unbound substances, the wells were incubated with HRP–streptavidin conjugate working solution for 30 min at 37 °C. Following additional washing steps, TMB substrate solution was added for color development, and the reaction was terminated using stop solution. The OD450 nm using a microplate reader to determine the MDK levels.

### 2.5. Cell Migration and Invasion Assay

In vitro cell migration and invasion were assessed using a Boyden chamber assay (Corning Incorporated, Corning, NY, USA) with an 8.0 μm polycarbonate membrane. For the migration assay, TSAIII-treated NPC cells (3 × 10^4^ cells per upper chamber) were suspended in serum-free medium and seeded into the upper chamber (without-Matrigel matrix coating membrane), while the lower chambers contained medium with FBS as a chemoattractant. For invasion assays, the membrane was pre-coated with 5% Matrigel matrix. After incubation for 18 h (migration assay) or 24 h (invasion assay), cells remaining on the upper surface were removed, and migrated/invading cells on the lower surface were fixed with methanol for 30 min and stained with 5% Giemsa (1:20) for 10 min. Cells were counted under a light microscope (200× magnification) in 5 randomly-selected fields for each treatment group.

### 2.6. Colony Formation

NPC cells (4 × 10^4^ cells/well) were treated with TSAIII (0~5 μM) for 7 days. Colonies were then fixed with methanol for 30 min and stained with Giemsa. Images were captured using a digital camera, and the number of colonies in each treatment group was determined.

### 2.7. Annexin V/PI Staining by Flow Cytometry

Cell death assays were performed using the Muse Annexin V & Dead Cell Assay Kit (Merck Millipore, Burlington, MA, USA). Cells (5 × 10^5^ per well) were seeded into 6-well plates and treated with TISAIII at various concentrations (0, 1, 3, and 5 µM) for 24 h. Cells were then collected, fixed, and stained in binding buffer with 5 µL of PI solution and 5 µL of FITC-conjugated Annexin V for 5 min in the dark. The cells were analyzed using flow cytometry.

### 2.8. Immunofluorescent Staining

NPC-BM and NPC-39 cells were immunofluorescently stained to examine MDK and ERβ localization. Cells were fixed with 4% paraformaldehyde for 20 min, permeabilized with 0.5% Triton X-100, and blocked with 5% bovine serum albumin for 1 h. Cells were incubated with primary antibodies against MDK and ERβ overnight at 4 °C, followed by incubation with fluorescent secondary antibodies for 1 h. Nuclei were counterstained with DAPI. Images were captured using an Axio Imager A2 microscope (ZEISS MicroImaging GmbH, Munich, Germany) at 400× magnification.

### 2.9. Human Chemokine Assay

The Human Chemokine Array Kit (ARY017; R&D Systems, MN, USA) was used to investigate TSAIII-targeting proteins. NPC-BM and NPC-39 cells were treated with TSAIII, lysed, and then incubated with nitrocellulose membranes coated with 31 different immobilized antichemokine antibodies. After washing, the membranes were incubated with streptavidin-HRP in each well of a 4-well dish and then visualized using a chemiluminescence detection system.

### 2.10. Data Collection and Differential Expression Analysis

Data regarding MDK expression in NPC tissues were obtained from the Gene Expression Omnibus (GEO) database (datasets GSE12452 and GSE53819). The MDK gene expression in NPC (tumor) and normal tissues (normal) was analyzed using data in the TNMplot database (https://tnmplot.com/analysis/; accessed on 30 October 2026).

### 2.11. Chromatin Immunoprecipitation (ChIP) Assay

The ChIP assay was performed as previously reported [21]. NPC-BM and NPC-39 cells were treated with TSAIII for 24 h. Formaldehyde cross-linking was used to trap DNA-protein interacting partners and isolate chromatin, followed by fragmentation by sonication to yield smaller DNA–protein fragments (about 100–200 bp). The fragments were immunoprecipitated with anti-Erβ antibody, incubated overnight at 4 °C, treated with proteinase K to digest the protein component, and the DNA purified using a DNA purification kit (Geneaid Biotech Ltd., New Taipei City, Taiwan). PCR was used to identify target protein (ERβ-DNA; MDK promoter) expression.

### 2.12. Western Blot Assay

Following TSAIII treatment, NPC-BM and NPC-39 cells were washed with ice-cold PBS and lysed with NETN buffer (protease inhibitor cocktail). Total-protein samples (15 μg) were separated by SDS–PAGE (10–12%), transferred onto PVDF membranes for 60 min, and then incubated with specific primary antibodies against ERα (1:1000), ERβ (1:1000), p-AKT-Thr308 (1:1000), p-AKT-Ser473 (1:1000), t-AKT (1:1000), MDK (1:1000), LMP-1 (1:1000), and β-actin (1:1000). After washing, the membranes were incubated with anti-mouse or anti-rabbit HRP-conjugated secondary antibody (1:10,000) for 60 min. Protein expression was visualized using enhanced chemiluminescence (Merck Millipore, Burlington, MA, USA) and quantified using an ImageQuant 800 Amersham Imager (Cytiva, Marlborough, MA, USA).

### 2.13. RT-qPCR Assay

Total RNA was isolated from TSAIII-treated NPC cells using TRIzol™ Reagent (Invitrogen, Carlsbad, CA, USA). The RNA (1 μg) was reverse-transcribed to cDNA using the GoScript Reverse Transcriptase kit (Promega Corporation, Madison, WI, USA). A StepOne Plus Real Time PCR System (Applied Biosystems, Thermo Fisher Scientific, Waltham, MA, USA) was then used to evaluate MDK mRNA levels, with GAPDH serving as the internal reference gene. The following primers were used: MDK-5′-CGCGGTCGCCAAAAAGAAAG-3′ (forward) and MDK-5′- TACTTGCAGTCGGCTCCAAAC-3′ (reverse); GAPDH, 5′-CATCATCCCTGCCTCTACTG-3′ (forward) and 5′-GCCTGCTTCACCACCTTC-3′ (reverse). The amplification conditions included the initial denaturation at 95 °C for 10 min, followed by 40 cycles of denaturation at 95 °C for 15 s and extension at 60 °C for 60 s. Relative transcript levels were calculated using the 2^−ΔΔCt^ method and normalized to GAPDH expression.

### 2.14. In Vivo Metastasis Assay, Immunohistochemistry, and Hematoxylin and Eosin Staining

Five-week-old male C.B17-SCID mice were purchased from the National Laboratory Animal Center (Taipei, Taiwan). The mice were housed under controlled environmental conditions at a room temperature of 22 ± 2 °C and a relative humidity of 60 ± 5%, with a 12 h light/dark cycle. All mice were provided ad libitum access to standard laboratory chow and water. The mice were randomly divided into 3 groups, as follows: Group 1, control (*n* = 5); Group 2, orally administered TSAIII (5 mg/kg; *n* = 5); Group 3, orally administered TSAIII (10 mg/kg; *n* = 5). Each mouse was injected via the tail vein with NPC-BM cells (1 × 10^6^ cells in 100 µL PBS). Seven days after injection, the mice were treated with TSAIII (5 or 10 mg/kg) by oral gavage twice per week for 10 consecutive weeks. At the end of the experimental period, mice were euthanized in a CO_2_ chamber. All euthanasia procedures were performed in accordance with institutional protocols and applicable veterinary guidelines. The lung, heart, liver, spleen, and kidneys were collected, fixed, and processed for histological analysis using hematoxylin and eosin (H&E) staining. For IHC analysis, lung tissue sections were incubated overnight at 4 °C with specific primary antibodies against Ki-67 (1:100), p-AKT (1:50), and MDK (1:100), followed by incubation with a secondary antibody (1:1000). Protein expression levels were visualized and evaluated under an optical microscope.

### 2.15. Statistical Analysis

All in vitro experiments were performed in triplicate (*n* = 3 independent biological replicates), whereas in vivo animal experiments included five mice per group (*n* = 5). Data are presented as the mean ± standard deviation (SD). Statistical analyses were performed using GraphPad Prism version 6.0 (GraphPad Software, San Diego, CA, USA). Differences among multiple groups were analyzed using one-way analysis of variance (ANOVA), followed by Tukey’s post hoc test for multiple comparisons. A *p* value < 0.05 or 0.01 was considered statistically significant.

## 3. Results

### 3.1. Effects of TSAIII on NPC Cell Proliferation, Apoptosis, and Metastatic Characteristics

**Figure 1:**
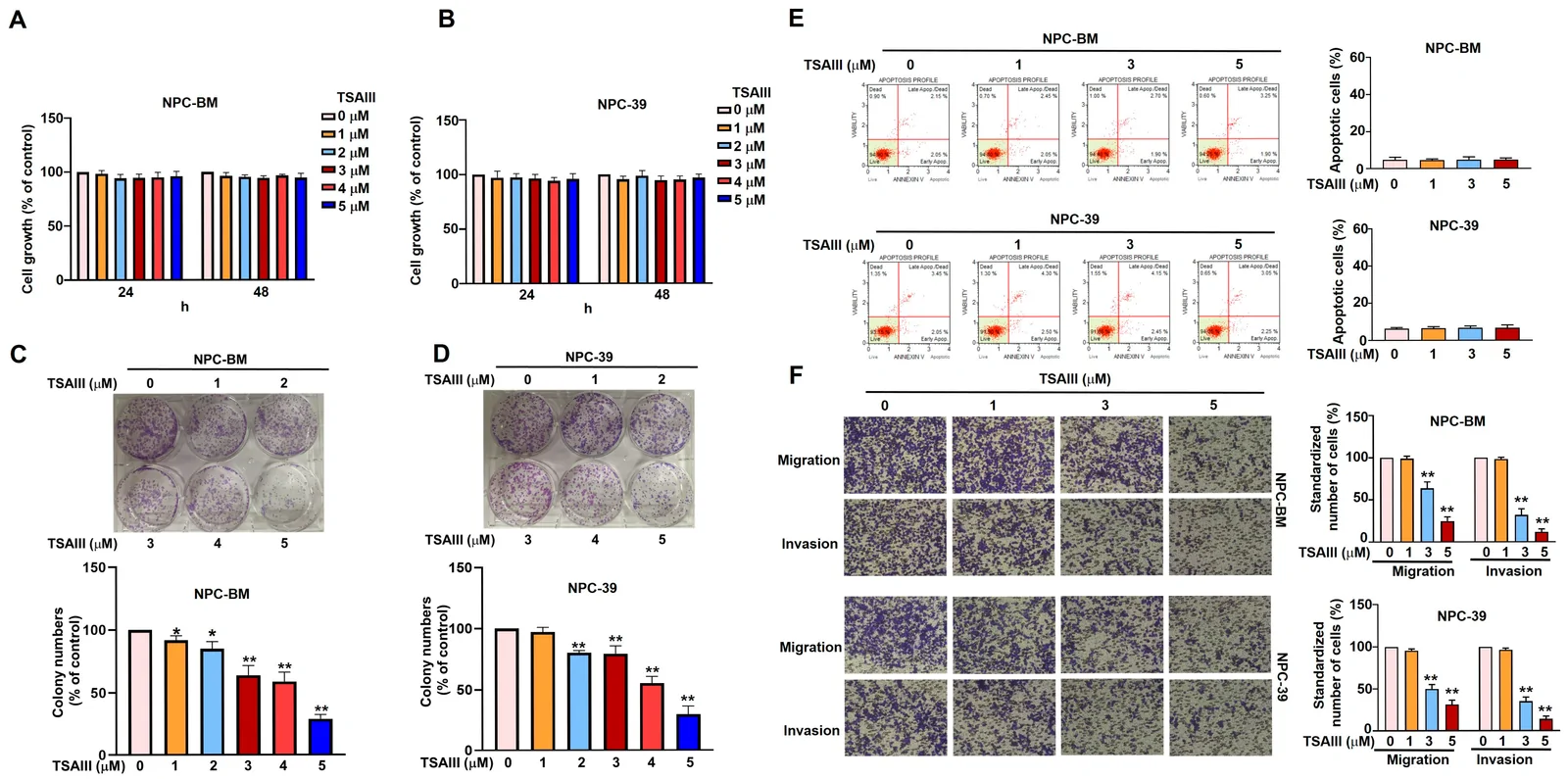
The effects of TSAIII on NPC cell growth, apoptosis, colony formation, migration, and invasion. (**A**,**B**) NPC-BM cells were treated with TSAIII (0 to 5 μM) for 24 and 48 h; cell viability was assessed using the MTT assay. (**C**,**D**) The proliferation rate of NPC-BM and NPC-39 cells was evaluated by colony formation assay after treatment with TSAIII (0–5 μM) for 7 days. (**E**) Apoptotic cells among NPC-BM and NPC-39 cells treated with TSAIII were detected using Annexin V/PI staining. (**F**) The migration and invasion abilities of NPC-BM and NPC-39 cells at concentrations of 1~5 μM. * *p* < 0.05 and ** *p* < 0.01 versus control.

Treatment with TSAIII at concentrations ranging from 0 to 5 μM for 24 and 48 h had no statistically significant effect on the proliferation of NPC-BM cells, as determined by cell viability assays (Figure 1A). Similarly, TSAIII treatment at the same concentration and duration did not significantly alter the growth of NPC-39 cells (Figure 1B). TSAIII treatment showed a significant reduction in colony formation in NPC-BM and NPC-39 cells, indicating that TSAIII may impair the long-term proliferative potential (Figure 1C,D). Flow cytometric analysis revealed that TSAIII did not significantly induce apoptosis in either NPC-BM or NPC-39 cells, even at the highest tested concentration (5 μM), suggesting that its cytostatic effects are not mediated through apoptotic pathways (Figure 1E). TSAIII markedly suppressed both the migration and invasion by NPC-BM and NPC-39 cells at concentrations ranging from 1 to 5 μM (*p* < 0.01), indicating a pronounced antimetastatic potential (Figure 1F). Collectively, these findings suggest that TSAIII effectively suppresses the migratory and invasive capacities of NPC cells. These findings highlight the potential utility of TSAIII for targeting metastasis in NPC cells.

### 3.2. Suppressive Effect of TSAIII on MDK Expression, Migration, and Invasion in NPC Cells

**Figure 2:**
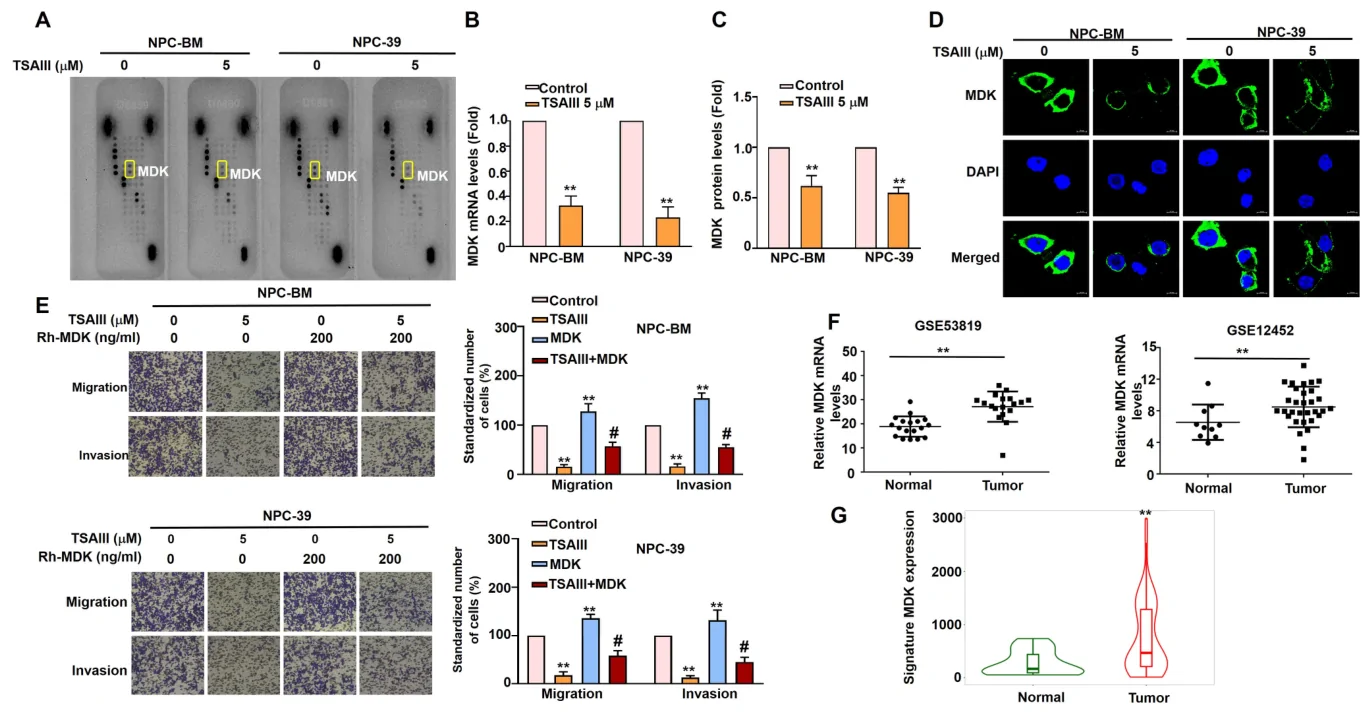
The effects of TSAIII on MDK expression and MDK-induced migration and invasion in NPC cells. (**A**) MDK expression in TSAIII (5 μM)-treated NPC cells. (**B**) qRT-PCR and (**C**) ELISA analysis of MDK mRNA expression and secretion level in NPC-BM and NPC-39 cells treated with TSAIII. (**D**) Immunofluorescence staining showing MDK protein expression (green) with nuclei counterstained with DAPI (blue). (**E**) The migratory and invasive abilities of both NPC cell lines were evaluated using in vitro migration and invasion assays. MDK expression levels in NPC tissues from (**F**) public datasets (GSE53819 and GSE12452) and (**G**) TNMplot database. ** *p* < 0.01 versus control. Scale bar = 10 μm. Original magnification 630×. # *p* < 0.05.

To investigate the molecular targets of TSAIII in NPC, a protein array was performed using NPC-BM and NPC-39 cell lines. The results showed that the MDK protein levels were markedly reduced following treatment with TSAIII (5 μM) (Figure 2A). Consistent with these findings, quantitative RT-PCR and ELISA analysis revealed a significant downregulation of MDK mRNA and secretion expression in both cell lines upon TSAIII treatment (Figure 2B,C). Immunofluorescence staining further confirmed a decrease in cytoplasmic MDK protein levels in TSAIII-treated cells (Figure 2D). To assess the functional consequences of MDK downregulation, Transwell migration and invasion assays were performed. Treatment with recombinant human MDK (Rh-MDK) at 200 ng/mL significantly increased cell migration and invasion by NPC-BM and NPC-39 cells. However, co-treatment with TSAIII partially reversed the pro-migratory and pro-invasive effects induced by Rh-MDK. These results suggest that TSAIII suppresses NPC cell motility and invasiveness, at least in part by inhibiting MDK expression (Figure 2E). To further explore the clinical relevance of MDK in NPC, public gene expression datasets were analyzed. MDK expression was significantly elevated in tumor tissues compared to normal tissues in both the GSE53819 and GSE12452 datasets (Figure 2F). In addition, TNMplot analysis demonstrated substantially higher MDK signature expression in tumor samples (Figure 2G). Collectively, these results suggest that MDK is overexpressed in NPC and contributes to tumor cell migration and invasion. TSAIII may exert its antimetastatic effects in part through the downregulation of MDK, highlighting MDK as a potential therapeutic target in NPC.

### 3.3. Suppressive Effects of TSAIII on Nuclear ERβ Expression and ERβ Binding to the MDK Promoter in NPC Cells

**Figure 3:**
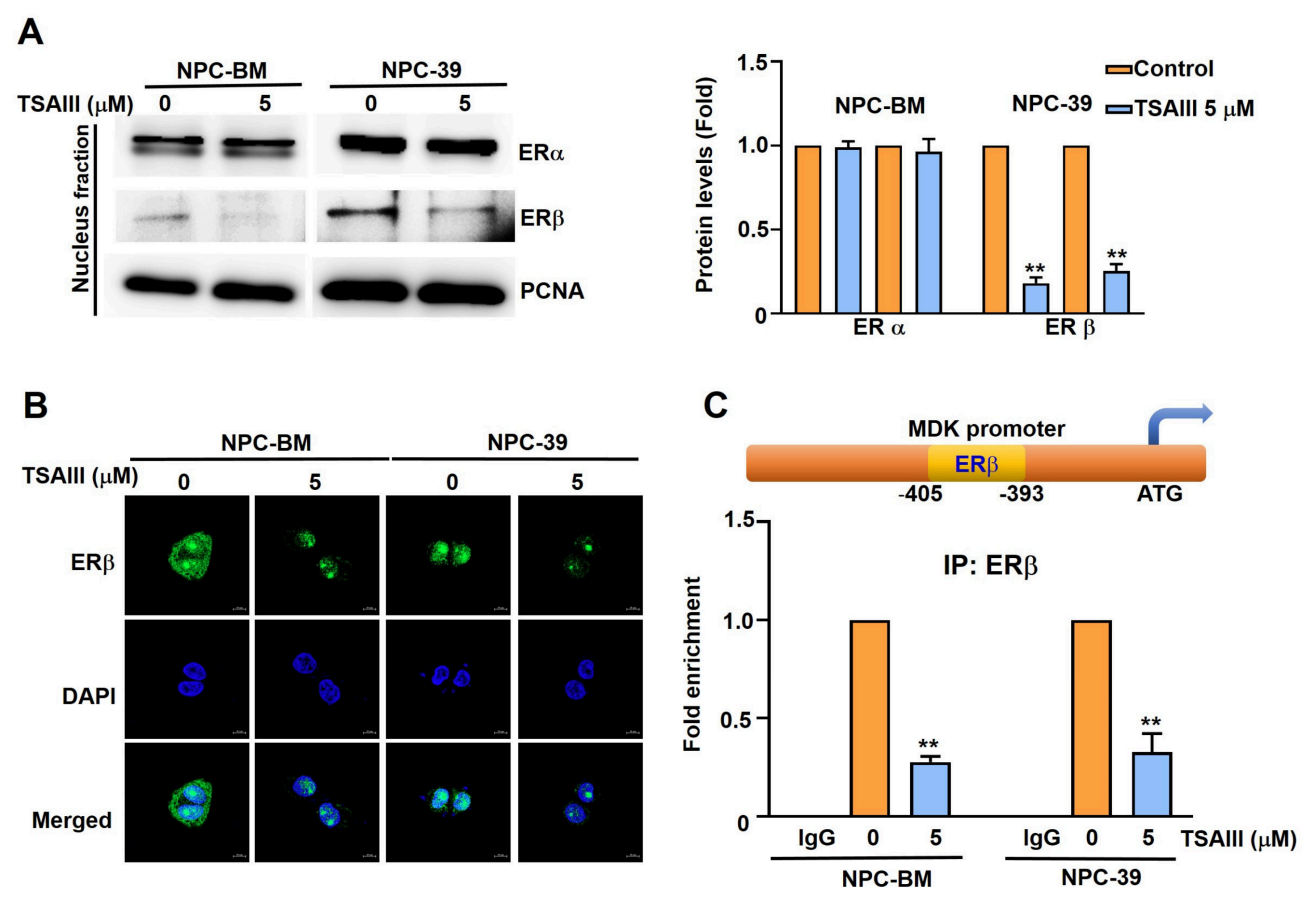
The effects of TSAIII on ERβ expression and binding activity of the MDK promoter in NPC cells. (**A**) Western blot analysis of ERα and ERβ expression in the nuclear fraction of both NPC cell lines treated with TSAIII (5 μM). (**B**) Immunofluorescence staining showing ERβ protein expression with nuclei counterstained with DAPI. (**C**) ChIP assay of ERβ binding to the MDK gene promoter region. ** *p* < 0.01 versus control. Scale bar = 10 μm. Original magnification 630×.

To explore the mechanism by which TSAIII regulates MDK expression, we examined the expression of estrogen receptor subtypes ERα and ERβ in the nuclear fraction of NPC cells. Western blot analysis revealed that nuclear ERβ but not ERα was markedly reduced in NPC-BM and NPC-39 cells following treatment with TSAIII (5 μM) (Figure 3A, left). Densitometric quantification confirmed a significant decrease in nuclear ERβ levels in both cell lines, while the ERα levels remained unchanged (Figure 3A, right). Consistent with these findings, immunofluorescent staining showed strong nuclear localization of ERβ in control cells, which was notably diminished after TSAIII treatment (Figure 3B). These results indicate that TSAIII selectively reduces nuclear ERβ levels in NPC cells. To determine whether ERβ directly regulates MDK transcription, we analyzed the MDK promoter sequence for potential ERβ binding sites. A putative ERβ-binding region was identified between positions −405 and −393 upstream of the MDK transcription start site (Figure 3C). ChIP assays using an anti-ERβ antibody demonstrated that ERβ was enriched at the MDK promoter in control cells, but this binding was significantly reduced in TSAIII (5 μM)-treated NPC-BM and NPC-39 cells (Figure 3C). Collectively, these results suggest that TSAIII suppresses MDK expression in NPC cells by downregulating nuclear ERβ and inhibiting ERβ binding to the MDK promoter, thereby disrupting ERβ-mediated transcriptional activation.

#### Inhibitory Effects of TSAIII on AKT-Activation–Mediated ERβ/MDK Expression in NPC Cells

**Figure 4:**
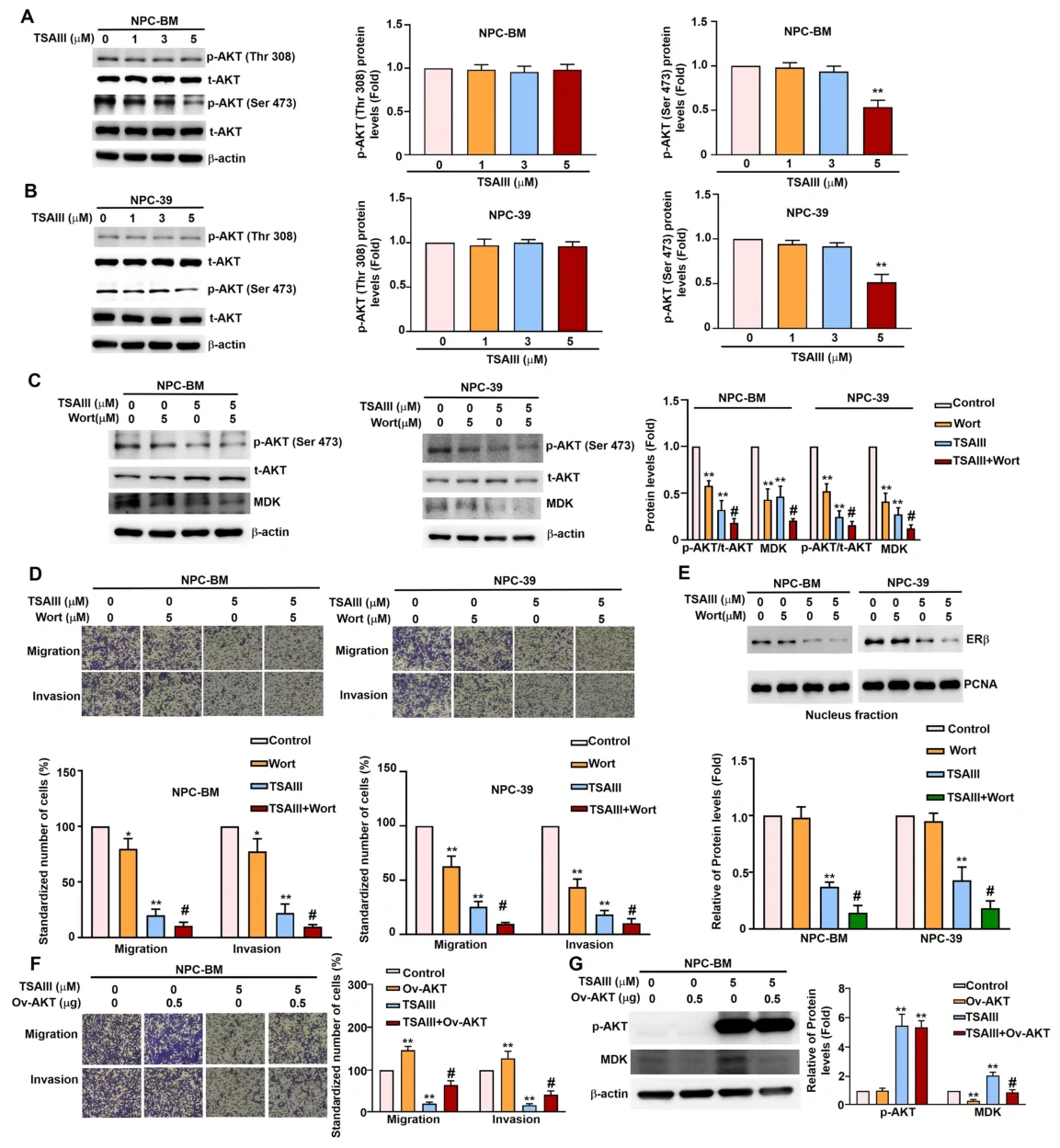
The effects of TSAIII on AKT activation via modulation of ERβ/MDK expression in NPC cells. (**A**,**B**) Western blot results (left panels) show protein bands for p-AKT (Thr308), p-AKT (Ser473), t-AKT, and β-actin in both NPC cell lines after treatment with varying concentrations of TSAIII (0, 1, 3, and 5 µM). The bar graph in the right panel quantifies the Western blot results. (**C**) Co-treatment with TSAIII and wortmannin in both NPC cell lines for 24 h, followed by Western blot analysis of t-AKT, p-AKT (Ser473), MDK, and β-actin. (**D**) Migration and invasion abilities of NPC-BM and NPC-39 cells. (**E**) Western blot analysis of ERβ expression in the nuclear fraction. PCNA as a nuclear marker. (**F**) Migration and invasion abilities of NPC-BM cells. (**G**) Transfection with constitutively active AKT plasmid (Ov-AKT) for 6 h, then with the TSAIII-treated NPC-BM cells added, followed by Western blot analysis. * *p* < 0.05, ** *p* < 0.01 versus control, and # *p* < 0.05 versus TSAIII-treated cells.

To examine whether TSAIII affects the AKT signaling pathway, we assessed the levels of total AKT (t-AKT) and phosphorylated AKT at two key activation sites (Thr308 and Ser473) in NPC-BM and NPC-39 cells treated with increasing concentrations of TSAIII (0, 1, 3, and 5 μM). At 5 μM, p-AKT(Ser473) phosphorylation was markedly decreased relative to the untreated control, whereas the total AKT remained unchanged, indicating selective inhibition of AKT activation rather than expression. Collectively, these results demonstrate that TSAIII suppresses AKT activity by reducing Ser473 phosphorylation without affecting total AKT or Thr308 phosphorylation. (Figure 4A,B). To determine whether p-AKT is involved in inhibiting ERβ/MDK expression and cell invasiveness, cells were co-treated with TSAIII and the AKT inhibitor (wortmannin) in NPC cells. The results showed that co-treatment inhibited p-AKT (Ser473) activity and the expression of MDK (Figure 4C) and nuclear ERβprotein (Figure 4D) and increased the anti-invasive effect in NPC cells (Figure 4E). To verify the role of AKT activation, TSAIII-treated NPC-BM cells were transfected with a constitutively active AKT plasmid (Ov-AKT), which restored AKT activity and reversed TSAIII-induced suppression of MDK expression (Figure 4F), migration, and invasion (Figure 4G). This finding suggests that TSAIII may exert its anti-invasive effects, at least in part, through inhibition of the p-AKT/ERβ/MDK expression signaling pathway in NPC cells.

### 3.4. TSAIII Suppresses LMP1-Driven Migration and Invasion in NPC-039 Cells by Targeting the p-AKT/ERβ/MDK Signaling Axis

**Figure 5:**
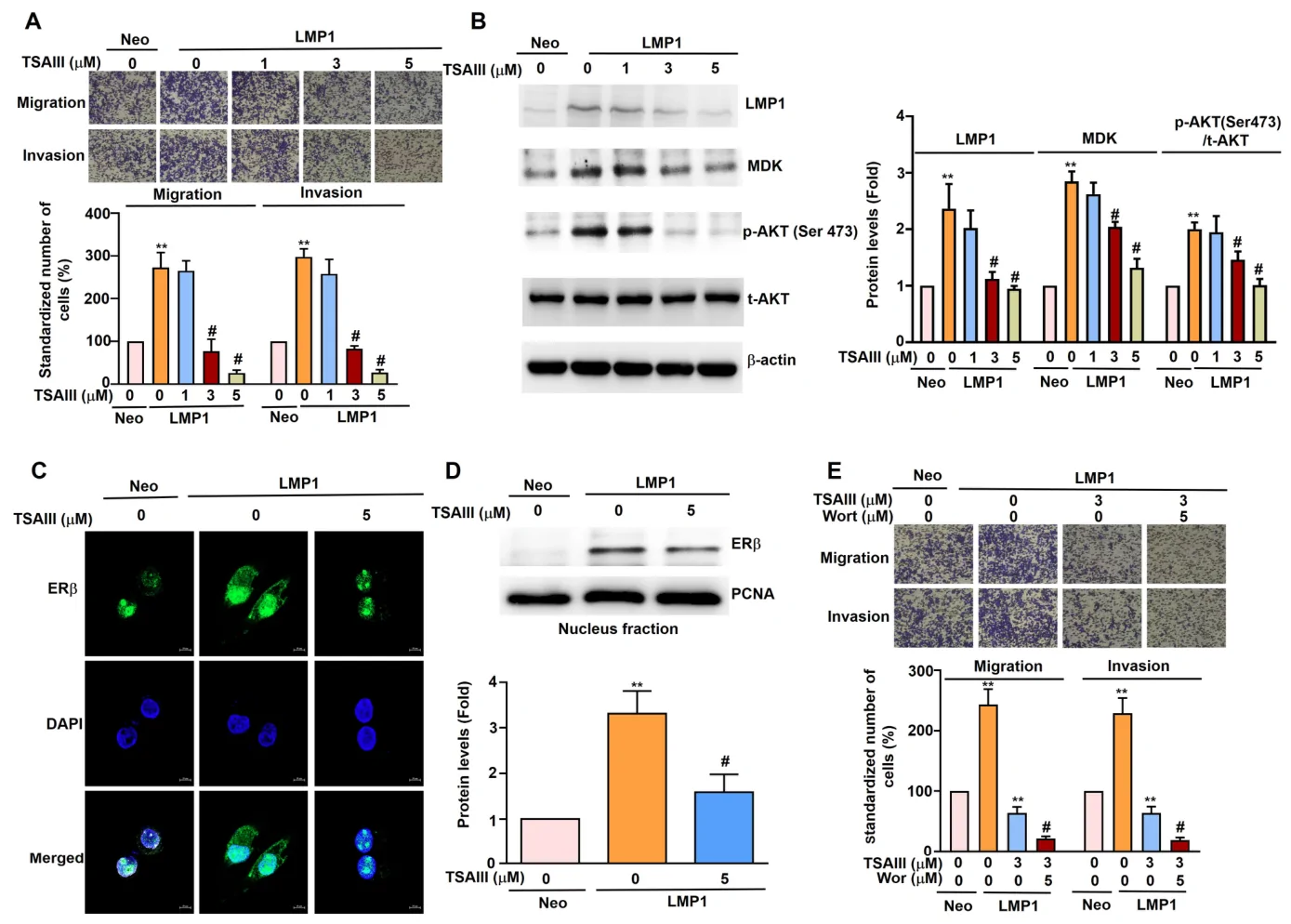
TSAIII inhibits LMP1-mediated ERβ/MDK expression and cell migration and invasion. (**A**) The migration and invasion of TSAIII-treated LMP1-NPC-39 cells. (**B**) Expression of LMP1, MDK, p-AKT and t-AKT were detected by Western blotting. (**C**) Immunofluorescence staining showing ERβ protein expression in TSAIII-treated LMP1-NPC-39 cells. (**D**) Nuclear protein expression of ERβ in TSAIII-treated LMP1-NPC-39 cells. ** *p* < 0.01 versus Neo and # *p* < 0.05 versus LMP1-treated cells. (**E**) The migration and invasion abilities of LMP1-NPC-39 cells co-treated with TSAIII and wortmannin. ** *p* < 0.01 versus Neo-NPC-39 cells and # *p* < 0.05 versus TSAIII+LMP1-NPC-39 cells. Original magnification 630×.

To examine the effect of TSAIII on LMP1-induced oncogenic characteristics, Neo- and LMP1-overexpressing NPC-39 cells were treated with increasing concentrations of TSAIII (0–5 μM). As shown in Figure 5A, LMP1 markedly increased cell migration and invasion, whereas TSAIII treatment significantly suppressed these effects in a dose-dependent manner. Western blot analysis demonstrated that TSAIII reduced the expression of MDK and the phosphorylation of AKT in LMP1-expressing NPC-39 cells without affecting the total AKT levels (Figure 5B). Immunofluorescent staining (Figure 5C) further revealed that LMP1 promoted the nuclear accumulation of ERβ, which was markedly diminished after TSAIII treatment. Consistent with this observation, Western blot analysis of nuclear fraction proteins showed a decrease in nuclear ERβ expression following TSAIII exposure (Figure 5D). Moreover, as illustrated in Figure 5E, co-treatment with TSAIII and the AKT inhibitor wortmannin exhibited enhanced inhibitory effects on LMP1-induced NPC-39 cell migration and invasion. Collectively, these findings indicate that TSAIII inhibits LMP1-mediated migration and invasion by suppressing the MDK/p-AKT/ERβ signaling pathway.

### 3.5. Suppressive Effects of TSAIII on NPC Cell Lung Metastasis

**Figure 6:**
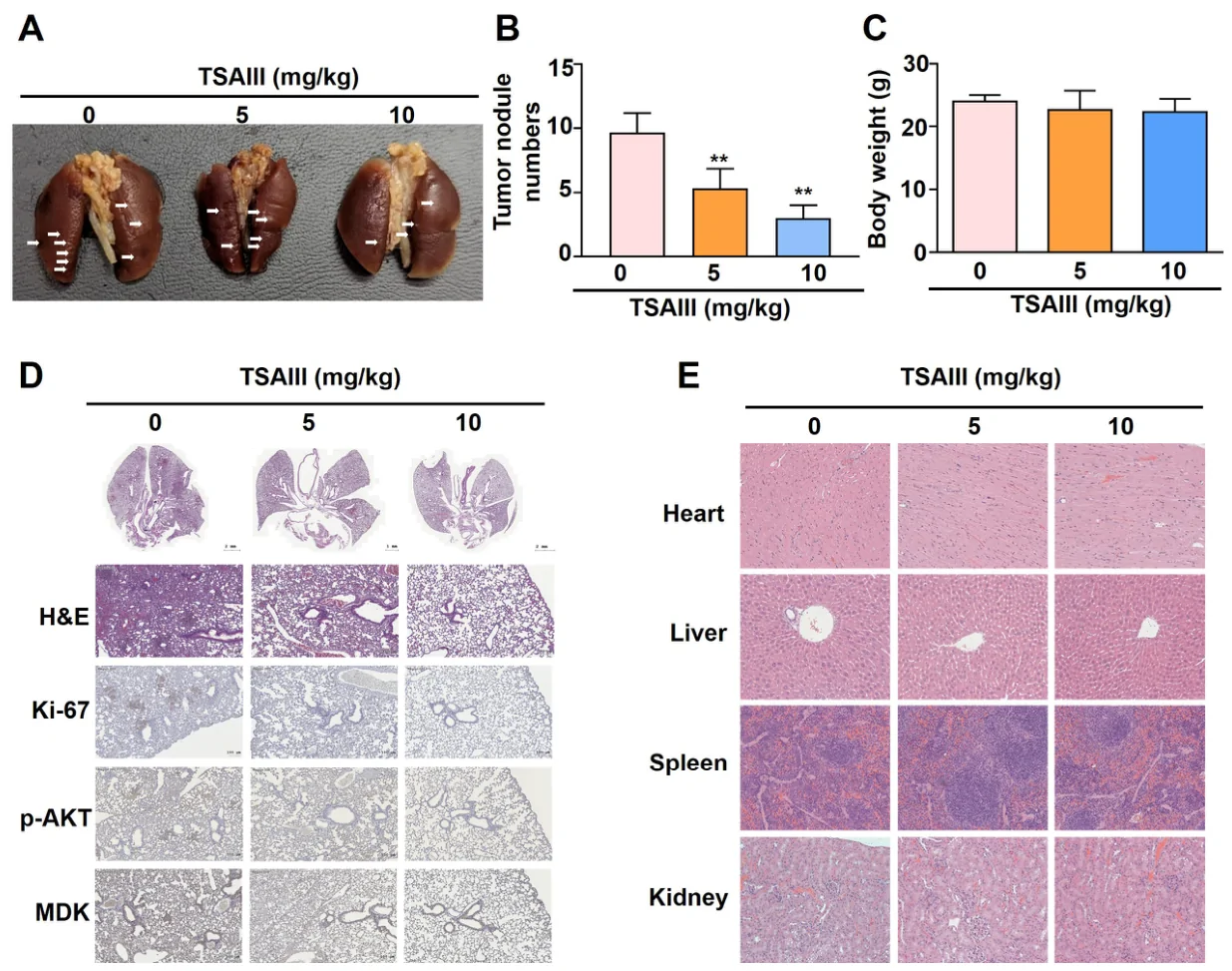
Effects of different doses of TSAIII on tumor growth in an NPC-BM mouse model. (**A**) The number of tumor nodules in the control group mice and TSAIII (5 and 10 mg/kg) group mice. (**B**) Bar graph quantifying the tumor nodule results in Panel (**A**). (**C**) Mouse body weights. (**D**) Full lung tissues and lung tumor tissues were detected by H&E and IHC staining of Ki-67, p-AKT, and MDK proteins in lung tumor tissues. (**E**) H&E staining of the different organ tissues (heart, liver, spleen, and kidney). ** *p* < 0.01 versus control. Scale bar = 2 mm and 100 μm. White arrows indicate tumor cells.

To evaluate the antimetastatic effects of TSAIII in vivo, NPC-BM-bearing mice were administered TSAIII at doses of 0, 5, and 10 mg/kg. As shown in Figure 6A, the number of visible metastatic tumor nodules in the lungs was markedly reduced in the TSAIII-treated groups compared to the control. Quantitative analysis (Figure 6B) confirmed a significant dose-dependent decrease in tumor nodules, while body weight remained unchanged across the groups (Figure 6C), indicating low systemic toxicity. Histological examination revealed that TSAIII treatment decreased tumor burden and Ki-67 staining (Figure 6D), suggesting reduced tumor cell proliferation. Moreover, the expression of phosphorylated AKT and MDK was notably diminished in lung metastatic lesions in TSAIII-treated mice. In addition, H&E staining of the heart, liver, spleen, and kidney (Figure 6E) revealed no apparent pathological changes, further supporting the safety of TSAIII administration. Collectively, these findings demonstrate that TSAIII effectively inhibits NPC lung metastasis in vivo through suppression of the MDK/p-AKT signaling pathway.

## 4. Discussion

In this study, we demonstrate that TSAIII selectively suppresses metastasis-related characteristics in NPC cells via downregulation of MDK/p-AKT/ERβ signaling, while exerting minimal effects on proliferation and apoptosis at doses up to 5 µM. These findings contribute to a new mechanistic understanding of the anticancer properties of TSAIII, complementing and extending previous work. Consistent with earlier reports, the action of TSAIII appears more potent in inhibiting migration, invasion, and colony-forming ability than in reducing cell viability. For example, in renal cell carcinoma lines treated with TSAIII (<8 µM), the cell viability and cell cycle distribution were not significantly changed, yet migration and invasion were suppressed, with an associated inhibition of PI3K/AKT signaling and upregulation of PTEN and miR-129-5p [20]. This finding mirrors our observation that even though apoptosis was not induced in NPC cells at similar (or lower) TSAIII concentrations, the metastatic properties were suppressed. Likewise, in human osteosarcoma cells, TSAIII inhibited migration and invasion without reducing cell growth or apoptosis, suggesting antimetastatic activity without cytotoxicity [22].

Accumulating evidence indicates that TSAIII can induce apoptosis and cell cycle arrest; however, these effects are typically observed at higher concentrations or in distinct tumor cells [14,23]. For example, in breast cancer cells, TSAIII was shown to induce G_2_/M phase arrest and apoptosis through activation of the ATM/Chk2 and p38 MAPK signaling pathways, accompanied by downregulation of cyclin B1, cdc2, and cdc25C [24]. In contrast, the absence of apoptosis or cell cycle arrest in NPC cell lines treated with up to 5 µM TSAIII suggests that these cells may exhibit reduced sensitivity to these effects at lower concentrations. Alternatively, this observation may indicate that TSAIII exerts antimetastatic activity at sub-cytotoxic doses, thereby highlighting a potentially favorable therapeutic agent. Our identification of MDK as a downstream target is well supported by studies in NPC and other cancers. For example, exosome miR-9 was shown to inhibit NPC tumor angiogenesis via targeting MDK and attenuating the PDK/AKT pathway [10]. This finding underscores the role of MDK not only in invasion/migration but also in angiogenesis, which is often intertwined with metastatic dissemination. In non-small-cell lung cancer, MDK promotes angiogenesis and metastasis, supporting EMT and NF-κB activation; targeting of MDK reduces migration, angiogenesis, and improves survival in animal models [25]. Thus, our findings that TSAIII downregulates MDK expression at the mRNA and protein levels, reduces MDK promoter activity via diminished ERβ binding, and suppresses p-AKT (Ser473) are consistent with the broader literature showing MDK/p-AKT as an axis that drives metastasis in NPC cells.

The role of estrogen receptors, particularly ERβ, in NPC is less well characterized, although ER signaling has been implicated in similar head and neck cancers [26]. While much of the literature has focused on ERα or combined ERα/ERβ effects, it may be fruitful to compare studies in other cancers. For example, ERβ has been shown to modulate AKT signaling and influence transcription of metastasis-associated genes in breast cancer [27]. Our results suggest a specific role for nuclear ERβ in driving MDK expression and that TSAIII reduces nuclear ERβ and its binding to the MDK promoter.

AKT is a proto-oncogene that is overactive in most cancers. AKT activation requires phosphorylation at two key residues: Thr308 (mediated by PDK1) and Ser473 (mediated by mTORC2). Full kinase activation depends on phosphorylation at both sites [28]; therefore, we examined both residues to determine whether TSAIII broadly suppresses AKT activation or selectively interferes with specific regulatory mechanisms. AKT pathway modulation is a recurring theme in the TSAIII literature. We observed that TSAIII reduces AKT Ser473 phosphorylation without affecting phosphorylation at Thr308—indicative of selective suppression of full activation. This finding is consistent with other studies reporting that TSAIII inhibits PI3K/AKT (and often downstream mTOR) signaling; for example, in taxol-resistant cancer lines (A549/Taxol, A2780/Taxol), Furthermore, in RCC cells, the antimigratory/invasive effects of TSAIII were partly mediated through the suppression of AKT signaling [20]. Future studies investigating MDK receptor engagement and downstream signaling are needed to fully elucidate this regulatory network. Thus, our observations in NPC enrich our understanding of the spectrum of tumors in which TSAIII inhibits the AKT pathway and suggest that suppression of Ser473 phosphorylation is a key nodal point for metastatic suppression rather than for inducing apoptosis. Substantial evidence supports a regulatory interaction between AKT and ER. AKT directly phosphorylates ERα at Ser167, enhancing its transcriptional activity in a ligand-independent manner. This phosphorylation event promotes ERα stability, nuclear localization, and recruitment of coactivators, thereby augmenting estrogen-responsive gene expression. For example, AKT phosphorylates ERα at Ser167 and increases ERα-mediated transcriptional activity [29]. Similarly, Khatpe et al. reported that growth factor-mediated activation of the PI3K/AKT pathway enhances ERα transactivation through phosphorylation-dependent mechanisms [30]. These findings establish AKT as an important upstream modulator of ERα function. Emerging evidence indicates that AKT signaling also regulates ERβ function. Although the interaction between AKT and ERα has been more extensively characterized, several studies suggest that ERβ activity can likewise be modulated by PI3K/AKT-dependent mechanisms. AKT has been shown to influence estrogen receptor transcriptional activity through phosphorylation-dependent signaling and co-regulator recruitment. In cellular models, activation of the PI3K/AKT pathway enhances ERβ-mediated transcriptional activity, whereas pharmacological inhibition of AKT attenuates ERβ-dependent reporter gene expression [31]. These findings suggest that ERβ is functionally responsive to upstream kinase signaling. Collectively, these studies support the biological plausibility that AKT acts as an upstream regulator of ER signaling, providing a mechanistic basis for the proposed AKT–ER regulatory axis.

Another important consideration is the interplay with viral oncogenes in NPC. Data showing that TSAIII suppresses LMP1-induced migration and invasion via MDK/p-AKT/ERβ are significant because LMP1 is a major driver of NPC metastasis and EMT. LMP1 has been shown to activate the PI3K/AKT signaling pathway in NPC [32]; the specific involvement of MDK in this pathway may be a novel finding in the present study. Thus, TSAIII appears to act both upstream (modulating promoter binding by ERβ) and downstream (AKT signaling) in the LMP1-driven oncogenic network, potentially making it a promising antagonist of EBV-mediated transformation. Our in vivo data showing reduced lung metastases, reduced MDK and p-AKT expression in lung lesions, and no overt systemic toxicity are consistent with studies in other cancers in which TSAIII has shown in vivo efficacy in breast cancer xenograft models [15] and osteosarcoma metastasis models [22]. Nevertheless, there are remaining questions and limitations. First, the role of apoptosis and cell cycle arrest in NPC at higher TSAIII concentrations or longer exposures needs further exploration to determine whether NPC cells are intrinsically less sensitive or delayed in those responses. Second, the potential off-target effects of TSAIII (e.g., on other transcription factors, coactivators, or alternative AKT upstream effectors) remain to be clarified. Third, bioavailability and pharmacokinetics in NPC models remain uncharacterized; TSAIII is relatively hydrophobic, and some studies indicate that delivery system improvements may increase its effect [14]. Third, although our in vivo findings support TSAIII’s antimetastatic potential, the intravenous injection model primarily assesses pulmonary colonization. Further studies using orthotopic NPC models are warranted to validate its efficacy. Lastly, the clinical relevance of ERβ in NPC patients, its expression pattern (nuclear vs. cytoplasmic) in tumors and metastases, and the expression of MDK in relation to ERβ and clinical outcomes deserve more study. Together with the current literature, our data reveal that TSAIII belongs to a growing class of agents with relatively selective antimetastatic effects that are mediated largely through suppression of the MDK/AKT axis and likely in cooperation with the modulation of transcriptional regulators such as ERβ. Our findings both corroborate and extend what is known about TSAIII in other cancers and define new molecular targets in NPC. Future efforts might focus on confirming these pathways in patient-derived xenografts, assessing combinatorial treatments (with AKT inhibitors, ER modulators), and improving the delivery and specificity of TSAIII.

Recent evidence has suggested the translational potential of TSAIII as an immunomodulatory anticancer agent, demonstrating immunomodulatory potential by suppressing A2AR/CREB/FoxP3 signaling, reducing CAR-Treg-mediated immunosuppression, and enhancing antitumor immune responses. Furthermore, TSAIII promotes CD8^+^ T-cell infiltration, improves responses to PD-1 blockade, and enhances central memory T-cell formation in preclinical models [33]. Another study found that TSAIII (KCT0002223), currently under clinical evaluation as a potential antiwrinkle agent, exhibits anti-inflammatory and antiphotoaging properties, with daily application for 12 weeks significantly reducing visible signs of facial aging, particularly wrinkle formation [34]. Collectively, these findings support TSAIII as a potential adjunct to cancer patient treatment, particularly immunotherapy. Furthermore, a clinical study suggested that baseline ^18^F-FDG PET/CT metabolic parameters may serve as valuable imaging biomarkers for predicting neoadjuvant chemotherapy (NAC) response in patients with NPC [35]. These findings highlight the clinical importance of treatment-response assessment in NPC and provide additional context for the potential translation of novel molecular therapeutic strategies. In this study, TSAIII targeting the p-AKT/ERβ/MDK signaling pathway may represent a promising therapeutic approach for suppressing NPC progression and metastasis. However, the relationship between this signaling pathway and ^18^F-FDG PET/CT imaging parameters remains unexplored. Further studies are needed to validate the immunomodulatory effects of TSAIII in NPC, assess its safety and therapeutic efficacy, and investigate its potential enhanced inhibitory effects when combined with conventional treatments.

## 5. Conclusions

**Figure 7:**
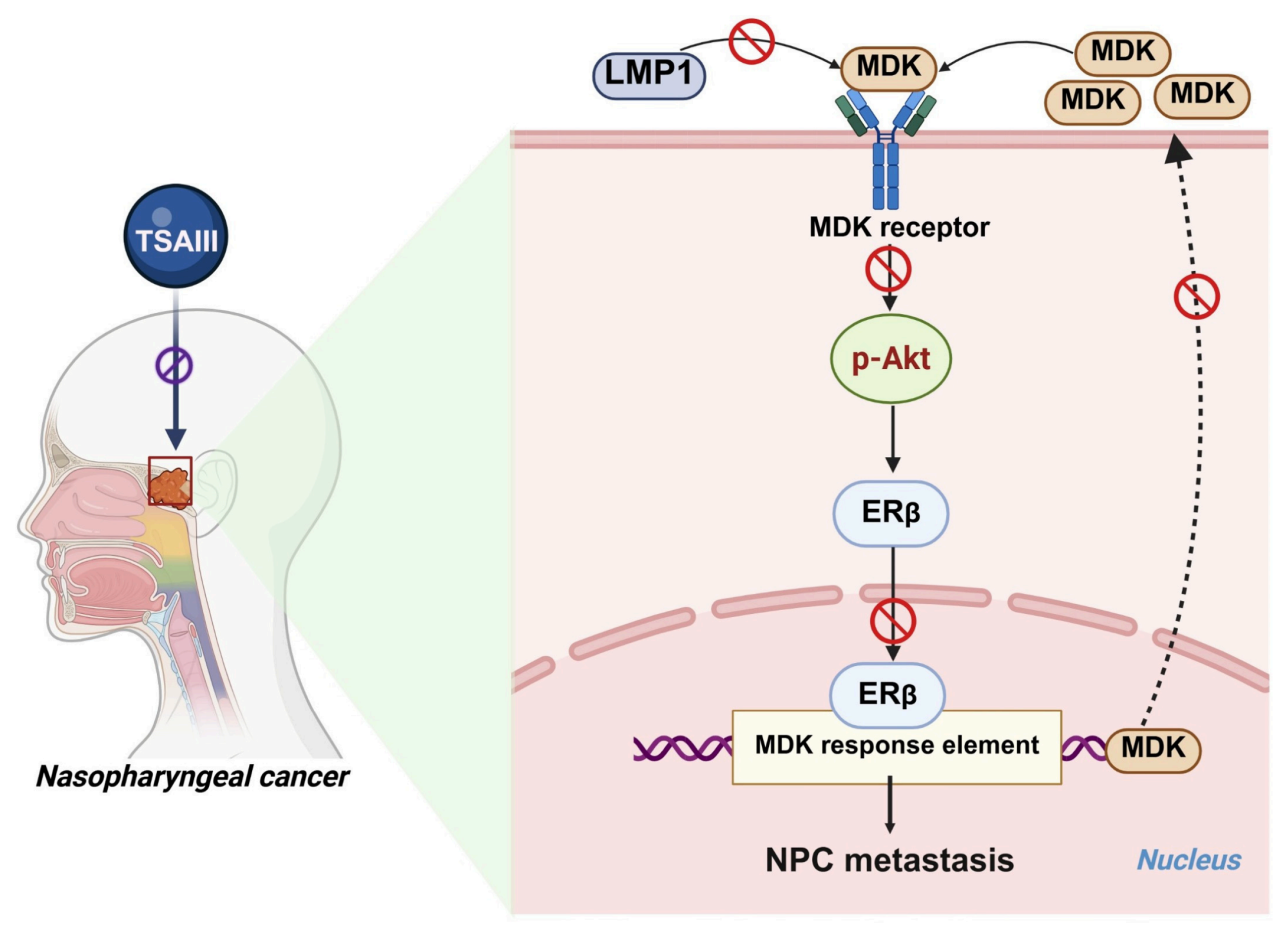
The mechanism by which TSAIII inhibits metastasis in nasopharyngeal cancer (NPC) by suppressing the p-AKT/ERβ-mediated MDK signaling pathway. TSAIII blocks LMP1-activated MDK receptor, inhibiting downstream p-AKT signaling. Activation of p-AKT promotes ERβ translocation into the cell nucleus, where it binds to the MDK response element. ERβ binding to the MDK response element activates MDK transcription, creating a positive feedback loop that leads to NPC metastasis.

Our findings suggest that TSAIII may suppress NPC cell migration and invasion without inducing significant cytotoxicity. Mechanistically, TSAIII treatment was associated with reduced AKT phosphorylation, ERβ nuclear localization, and MDK transcription, suggesting the potential involvement of LMP1-associated p-AKT/ERβ/MDK signaling. However, potential regulatory feedback mechanisms remain to be elucidated. In vivo, TSAIII reduces pulmonary metastases without detectable organ toxicity (Figure 7), highlighting its potential as a promising antimetastatic therapeutic candidate for NPC. Further mechanistic and preclinical studies are warranted to validate these preliminary findings.

## Data Availability

The data that support the findings of this study are available from the corresponding author upon reasonable request.

## Abbreviations

BSA	Bovine serum albumin
ChIP	Chromatin immunoprecipitation
EBV	Epstein–Barr Virus
ERK1/2	Extracellular signal-regulated kinase 1/2
ERα	Estrogen receptorα
ERβ	Estrogen receptorβ
FBS	Fetal bovine serum
IHC	Immunohistochemical
JNK1/2	c-Jun N-terminal kinase 1/2
p38MAPK	p38 mitogen-activated protein kinases
LMP1	Latent membrane protein 1
MDK	Midkine
NPC	Nasopharyngeal carcinoma
qRT-PCR	Quantitative real-time reverse transcription–polymerase chain reaction
SDS-PAGE	Sodium dodecyl sulfate–polyacrylamide gel electrophoresis
siRNA	Small interfering ribonucleic acid
TCGA	The Cancer Genome Atlas
TSAIII	Timosaponin AIII
